# Emerging Phytochemicals for the Prevention and Treatment of Head and Neck Cancer

**DOI:** 10.3390/molecules21121610

**Published:** 2016-11-24

**Authors:** Santosh K. Katiyar

**Affiliations:** 1 Comprehensive Cancer Center, University of Alabama at Birmingham, Birmingham, AL 35216, USA; skatiyar@uab.edu; Tel.: +1-205-975-2608; 2 Nutrition and Obesity Research Center, University of Alabama at Birmingham, Birmingham, AL 35216, USA; 3 Birmingham Veterans Affairs Medical Center, Birmingham, AL 35233, USA

**Keywords:** head and neck cancer, epidermal growth factor receptor, phytochemical, cell migration, tumor growth

## Abstract

Despite the development of more advanced medical therapies, cancer management remains a problem. Head and neck squamous cell carcinoma (HNSCC) is a particularly challenging malignancy and requires more effective treatment strategies and a reduction in the debilitating morbidities associated with the therapies. Phytochemicals have long been used in ancient systems of medicine, and non-toxic phytochemicals are being considered as new options for the effective management of cancer. Here, we discuss the growth inhibitory and anti-cell migratory actions of proanthocyanidins from grape seeds (GSPs), polyphenols in green tea and honokiol, derived from the *Magnolia* species. Studies of these phytochemicals using human HNSCC cell lines from different sub-sites have demonstrated significant protective effects against HNSCC in both in vitro and in vivo models. Treatment of human HNSCC cell lines with GSPs, (−)-epigallocatechin-3-gallate (EGCG), a polyphenolic component of green tea or honokiol reduced cell viability and induced apoptosis. These effects have been associated with inhibitory effects of the phytochemicals on the epidermal growth factor receptor (EGFR), and cell cycle regulatory proteins, as well as other major tumor-associated pathways. Similarly, the cell migration capacity of HNSCC cell lines was inhibited. Thus, GSPs, honokiol and EGCG appear to be promising bioactive phytochemicals for the management of head and neck cancer.

## 1. Introduction

Head and neck squamous cell carcinoma (HNSCC) is diagnosed in >40,000 individuals in the United States each year and it is responsible for approximately 20,000 deaths annually [1,2]. Over the past 30 years there has been only a marginal improvement in the survival rate [3,4], despite advances in surgical and medical therapies during the past 10 years [5,6]. Most patients with early stage HNSCC can be effectively treated with single modality treatment using local radiation or surgical intervention and the use of platinum-based chemotherapy as a radiosensitizing agent has been shown to improve treatment outcomes. The currently available therapies, including conventional chemotherapy and surgical resection, are, however, often associated with impairment of speech and swallowing functions. Moreover, the effective use of chemotherapy is problematic due to considerable toxicities and drug resistance [7,8,9,10]. A major breakthrough has been the introduction of targeted biologic therapies. Considerable evidence, derived from both the laboratory and the clinic, has implicated epidermal growth factor receptor (EGFR) and its signaling in the development of HNSCC tumors. The overexpression of EGFR in approximately 90% of HNSCC tumors [11,12,13] and the significant association of its overexpression with poor prognosis identified EGFR as a promising target for the treatment of HNSCC. Cetuximab, a chimeric antibody that targets EGFR, has been approved by the FDA for the treatment of HNSCC and erlotinib, an EGFR-targeting small molecule tyrosine kinase inhibitor, is currently under evaluation in clinical trials for HNSCC [14]. The emerging issue of the development of resistance to biologic therapies limits the use of these drugs, however. Thus, the development of novel strategies or complementary and alternative therapies are needed. In terms of treatment, approaches that can be used in combination with available treatment modalities to lower the doses of toxic drugs and to overcome drug resistance are required. An optimal treatment strategy would ensure treatment efficacy, reduce toxicity and improve the quality of life in the patients suffering from HNSCC. Given the difficulties associated with management of HNSCC tumors and the significant associated morbidities, the development of chemopreventive strategies is an attractive and important alternative even with the advent of novel biologic therapies [15]. Chemopreventive agents are defined as having the ability to arrest, reverse or slow carcinogenesis at its earliest stages. Because chemopreventive strategies must be used consistently over the life-time of the individual, they must not only be effective but also have a low toxicity profile as well as being affordable and easy to use.

Natural products obtained from plants, herbs and shrubs have been used for thousands of years in traditional systems of medicine, such as the Chinese, the Unani the Indian Ayurvedic systems of medicine. Bioactive components from members of the plant kingdom that are non-toxic at effective doses offer both promising new options for the development of effective chemotherapeutic or adjuvant therapy for conventional cytotoxic therapies and the development of chemopreventive approaches. Thus, considerable attention is being paid to analysis of the effects on HNSCC growth and cell invasion of those phytochemicals that have low toxicity. This article provides information on selected phytochemicals that have shown promising chemopreventive or chemotherapeutic effects on the growth of HNSCC cells and their migration.

Two of the phytochemicals described in this review (grape seed proanthocyanidins and (−)-epigallocatechin-3-gallate) are polyphenols. Polyphenols are one of the largest and most ubiquitous groups of phytochemicals. They are subdivided according to their molecular structure, including the number of aromatic phenol rings and the structural elements that link these rings. The polyphenols, which includes phenolic acids, stilbenes, lignans and flavonoids, are ubiquitous in the human diet, with flavonoids and phenolic acids being the most common. The richest dietary sources of polyphenols are fresh fruits and vegetables (including legumes), green tea and red wine. In recent years, there has been a growing awareness of the possibility that a lower incidence of cancer in some populations may be associated with the consumption of certain nutrients and especially a diet rich in polyphenols.

**Figure 1:**
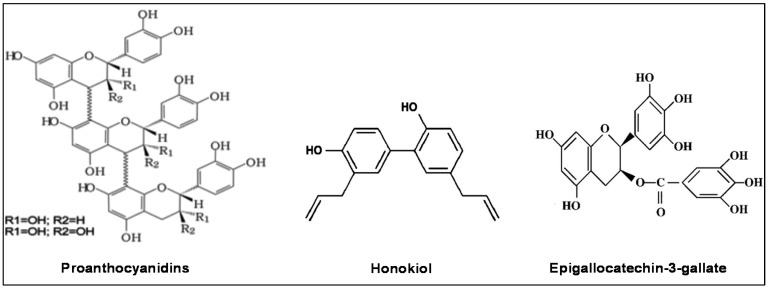
Molecular structures of phytochemicals that have shown promise for therapeutic use in the management of head and neck cancer. Proanthocyanidins are shown as polymers of catechin monomers.

Systematic studies of the potential health benefits of naturally occurring polyphenols have included investigations of their protection against oxidative stress, inflammation, diabetes, cardiovascular disease, asthma, and aging, as well as their anti-cancer properties. The combination of their wide range of potential chemopreventive characteristics, their efficacy and their lower associated toxicity as compared to conventional therapeutic drugs has drawn particular interest. The intention of this article is to outline the anti-HNSCC effects of some promising phytochemicals, including polyphenols that have shown encouraging protective effects against the growth and metastasis of HNSCC based on in vitro studies and/or preclinical animal models. The phytochemicals reviewed are: (i) grape seed proanthocyanidins; (ii) honokiol; and (iii) (−)-epigallocatechin-3-gallate (EGCG), a major component of green tea polyphenols. The molecular structures of these agents are shown in Figure 1.

## 2. Promising Phytochemicals

### 2.1. Grape Seed Proanthocyanidins (GSPs)

Proanthocyanidins are widely distributed in fruits, vegetables, seeds, nuts, and bark and are found in some flowers. They are a class of phenolics that take the form of oligomers or polymers of polyhydroxy flavan-3-ol units, such as (+)-catechin and (−)-epicatechin monomers [16]. Grapes are rich in polyphenols, with approximately 60% to 70% of the grape polyphenols being found in the seeds, which are a plentiful byproduct of the industrial production of grape juice and wine. The seeds contain a larger fraction of proanthocyanidins, which are composed of dimers, trimers, tetramers, and oligomers of monomeric catechins or epicatechins, than the other plant parts [17,18,19]. These grape seed proanthocyanidins (GSPs) have been shown to have cytotoxic activity against various cancer cell lines, including those from several different types of cancer [20,21,22] with no significant toxic effects on normal cells [23,24]. Various studies have shown that GSPs possess antioxidant and anti-inflammatory as well as anti-carcinogenic activities [25,26].

#### 2.1.1. GSPs Inhibit the Growth of HNSCC Cells

Numerous studies have documented the anti-cancer activity of GSPs in various preclinical models [14,20,27,28,29,30,31]. The author’s laboratory examined the therapeutic effects of GSPs using different HNSCC cell lines in both in vitro and in vivo models [32]. It was found that treatment with GSPs significantly reduced the viability, and induced apoptosis, of human HNSCC cells, including HNSCC cell lines derived from the oral cavity (SCC1), larynx (SCC5), pharynx (FaDu) and tongue (OSC19). In these studies, the GSPs did not exhibit significant toxicity against non-neoplastic human bronchial epithelial cells under identical conditions. Thus, these results suggest that GSPs may have a beneficial therapeutic effect on HNSCC. 

Inhibition of cell cycle progression in cancer cells has been identified as an effective strategy for the control of cancer growth [33,34]. In vitro experiments indicated that treatment of HNSCC cells with GSPs resulted in arrest of cell cycle progression at the G0-G1 phase, which indicates that one of the mechanisms by which GSPs may act to inhibit the proliferation of HNSCC cells is inhibition of cell cycle progression. The GSPs-induced arrest of the cell cycle at the G0/G1 phase was associated with a reduction in the levels of cyclins and cyclin-dependent kinases (Cdks) in the SCC1 and OSC19 cells, which further suggests that the GSPs can act to disrupt the uncontrolled cell cycle progression in HNSCC cells. In addition, GSPs treatment resulted in a restoration of the levels of tumor suppressor proteins, such as Cip1/p21 and Kip1/p27, in the HNSCC cells. The GSPs-induced upregulation of Cip1/p21 in the HNSCC cell line appears to be mediated through a p53-independent pathway. Arrest of the cell cycle at the G1 phase provides an opportunity for cells to either undergo repair mechanisms or enter pathways leading to apoptosis. Treatment of HNSCC cells with GSPs resulted in significant induction of apoptosis. Apoptosis plays a crucial role in eliminating the mutated neoplastic and hyper-proliferating neoplastic cells and is, therefore, considered of importance in protecting against cancer progression [35]. 

**Figure 2:**
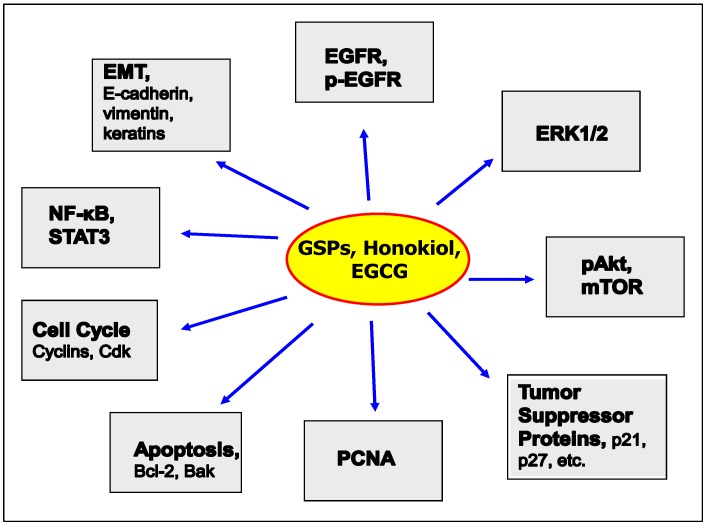
Molecular targets of phytochemicals (GSPs, honokiol and green tea polyphenols) in the prevention or treatment of HNSCC. Studies have shown that the major molecular targets of these phytochemicals on HNSCCs are EGFR, EMT, cell cycle regulators, apoptotic proteins and PCNA, etc. as shown.

The induction of apoptosis is a tightly regulated and complex process. Induction of apoptosis of tumor cells can involve the upregulation of pro-apoptotic proteins and/or downregulation of anti-apoptotic proteins. Proteins in the Bcl-2 family can play a pivotal role in determination of the fate of the cells as they can either promote cell survival or promote apoptotic cell death depending on circumstance [36,37]. Treatment of SCC1 and OSC19 cells with GSPs resulted in a decrease in the levels of anti-apoptotic proteins and a simultaneous increase in the pro-apoptotic proteins, as well as release of cytochrome c from mitochondria. Release of cytochrome c leads to the activation of caspases and the enzyme poly ADP ribose polymerase (PARP) thereby leading to apoptosis. Collectively, these results are consistent with the effects of the GSPs on HNSCC cell viability and apoptosis and suggest possible mechanisms of action of the GSPs against HNSCC cells (Figure 2).

As described in the Introduction to this article, EGFR is overexpressed in most cases of HNSCC and its inhibition is considered as a promising therapeutic approach in head and neck cancer. The stimulation of EGFR in HNSCC results in cell proliferation, a reduction in apoptosis and increased tumor cell metastasis, and is associated with poor clinical outcome in this malignancy [11,12,13]. Treatment of SCC1 and OSC19 cells with GSPs decreased the expression levels of total EGFR as well as the levels of phosphorylated EGFR and also inhibited the levels of the EGFR downstream target, ERK1/2. Thus it appears that inhibition of cell viability by GSPs treatment is mediated, at least in part, through the downregulation of the EGFR/ERK signaling cascade. Similar effects were observed when HNSCC cells were treated with erlotinib, an inhibitor of EGFR. Although EGFR is of great importance in HNSCC, it should be noted that other factors and molecular targets also play roles in the growth, progression and metastasis of HNSCCs and that the ability of phytochemicals to target these other pathways will be of significance in consideration of their use as complementary or alternative strategies.

The in vitro cell culture models described above are a good system for preliminary screening of the effects of chemotherapeutic agents; however, testing in preclinical models is necessary to move the field forward. The outcomes of treatment must be verified in vivo using animal models prior to potential consideration of their application in humans. Thus, tumor xenograft models were used to further determine the anti-carcinogenic effects of GSPs. The GSPs were administered in the diet of the animals as this approach has proven effective in other types of cancers [14,20,27,28,29,30,31,38]. Animal studies have shown that dietary administration of GSPs inhibits the growth of HNSCC tumor xenografts without any apparent sign of toxicity in the athymic nude mice. The effect of dietary GSPs was also tested on different biomarkers of interest in an effort to evaluate the clinical efficacy of GSPs as well as to elucidate the mechanisms of action. In this context, the inhibitory effect of dietary GSPs on the growth of tumor xenograft was found to be associated with: (i) control of cell cycle regulation; and (ii) induction of apoptotic cell death of tumor cells, which were associated with the observation of effects on the proteins of Bcl-2 family and activation of caspase-3 in tumor xenograft samples at the termination of the animal experiments. Thus, the results of this study showed for the first time the chemotherapeutic efficacy of GSPs on the growth of head and neck cancer cells in vitro and tumor xenograft growth in vivo. 

#### 2.1.2. GSPs Inhibit the Migration Potential of HNSCC Cells

The migration capacity or metastatic potential of HNSCC cells is a significant contributing factor to the high mortality rate of this malignancy as well as the difficulties associated with its treatment. As advances in surgical and medical therapies for HNSCC have resulted in only a modest improvement in the mortality rate [3,4,5,6], elucidation of the molecular targets and signaling mechanisms essential for metastasis and identification of approaches that minimize the migration and metastatic behaviors of cancer cells is of the highest priority. Attempts have been made to assess the anti-migration ability of GSPs on HNSCC cells. As HNSCCs arise from different sub-sites, the author’s laboratory has tested the invasive potential of HNSCC cell lines developed from the oral cavity, larynx, and pharynx. The cell lines derived from tumors from these different sites exhibited greater invasive potential than normal bronchial epithelial cells. The cell migration capacity of the OSC19 cell line, which originated from the tongue, was found to be greater than the other cell lines obtained from oral cavity, larynx and pharynx (SCC1, SCC5 and FaDu). Treatment with GSPs was found to inhibit the migration of OSC19 cells in a dose-dependent manner [39]. The OSC19 cells overexpress EGFR and this inhibitory effect of GSPs on cell migration was associated with the induction of reduced levels of EGFR expression [39]. The concept that the inhibition of EGFR by GSPs may contribute to the inhibition of cell migration capacity of these cells is supported by the finding that treatment of the OSC19 cells with gefitinib or erlotinib, small molecule inhibitors of EGFR, also resulted in a reduction in the cell migration ability. Nuclear factor-kappaB (NF-κB) has been identified as an important regulator of cancer cell invasion, metastasis and angiogenesis and is a downstream target of EGFR [40,41,42]. Treatment of OSC19 cells with GSPs resulted in downregulation and inactivation of the NF-κB pathway. Furthermore, treatment of HNSCC cells with an inhibitor of NF-κB (caffeic acid phenethyl ester) inhibited the invasion of HNSCC cells. NF-κB-targeted proteins, such as matrix metalloproteinases (MMPs), cyclooxygenase-2 (COX-2), inducible nitric oxide synthase (iNOS) and vascular endothelial growth factor (VEGF), also have been implicated in cancer cell migration. Treatment of OSC19 cells with GSPs reduced the levels of these NF-κB-targeted proteins. These data support the existing evidence that NF-κB has a role in determining the migratory potential of HNSCC cells, and that the inhibitory effect of GSPs on cell migration is mediated, at least in part, through inactivation of NF-κB. These studies also revealed that the GSPs inhibition of the migratory potential of OSC19 cells is associated with the inhibition of ERK1/2, suggesting a possible involvement of the ERK1/2-NF-κB pathway in the effects of GSPs on migration of HNSCC cells. 

The epithelial-mesenchymal transition (EMT) in cancer cells plays a major role in determining the metastatic potential of epithelial tumors. EMT can render tumor cells migratory and invasive through its effects on all involved stages, including invasion, intravasation and extravasation [43,44]. The process of EMT is coordinated primarily by the loss of epithelial biomarkers such as *E*-cadherin and certain cytokeratins, concomitant with the acquisition of mesenchymal phenotypes or biomarkers, e.g., vimentin, *N*-cadherin and fibronection. Treatment of OSC19 cells with GSPs resulted in not only the downregulation of mesenchymal biomarkers but also reactivation of epithelial biomarkers. This suggests that GSPs have the ability to reverse the EMT process in HNSCC cells and this reversal may be considered as one of the possible mechanisms through which GSPs inhibit the migratory potential of HNSCC cells. Notably, NF-κB, which has been identified as a target of HNSCC cells, as described above, has been identified as an important regulator of the EMT in cancer cells [40,41,42].

**Figure 3:**
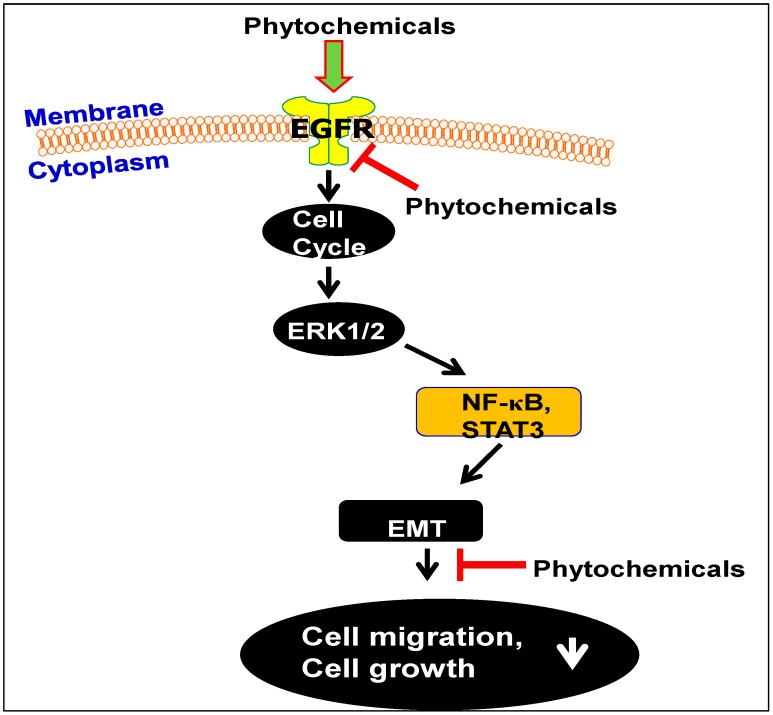
Summary of the action of emerging phytochemicals for the prevention and therapy of HNSCC. Notably, the phytochemicals that were selected based on their efficacy in inhibiting proliferation of HNSCC cells or induction of their apoptosis, all inhibited the expression levels of EGFR in human HNSCC cells. Overexpression of EGFR plays a critical role in cancer cell growth and cancer cell migration/invasion. In general, the inhibition of cell growth and migration capacity of the HNSCC cells by the GSPs, GTPs and honokiol is mediated through their effect on the cell cycle regulatory proteins, EMT and NF-κB signaling pathway.

These data suggest that inhibition of the migratory potential of HNSCC cells by GSPs involves the: (i) inhibitory effect of GSPs on EGFR overexpression; (ii) inhibitory effect of GSPs on the activation of the ERK1/2 proteins and inactivation of NF-κB; and (iii) reversal of the EMT, as summarized in Figure 2 and Figure 3. Sun et al. [45] have examined the effect of GSPs on the migration ability of head and neck cutaneous squamous cell carcinoma cells (SCC13) using in vitro cell invasion assays. They reported that treatment of the SCC13 cells with GSPs resulted in inhibition of cell invasion which was associated with a reduction in the levels of EGFR in the tumor cells, and a reversal of the EMT process. Collectively these data suggest that GSPs can be developed as a complementary and alternative medicine for the prevention of invasion/metastasis of HNSCC cells. However, more in vivo studies are needed to verify the therapeutic potential of this phytochemical. 

### 2.2. Honokiol and HNSCC

Honokiol (molecular formula, C_18_H_18_O_2_) is a biphenolic plant product that is isolated from the bark and leaves of plants of the *Magnolia* spp. Honokiol has been demonstrated to have anti-carcinogenic, anti-inflammatory, anti-oxidative, and anti-angiogenic effects as well as inhibitory effects on the malignant transformation of skin papillomas to carcinomas [46,47,48,49,50]. This phytochemical affects multiple molecular and cellular targets including NF-κB, STAT3, EGFR, cell survival signaling, and cell cycle check points as well as expression of COX-2 and other inflammatory mediators. Its chemopreventive and/or therapeutic effects have been determined using multiple different tumor models [46,47,48,49,50,51].

In the context of its effects in HNSCC, Singh et al. [51] examined the therapeutic efficacy of honokiol using both in vitro and in vivo preclinical animal models. Honokiol reduced the viability, and induced apoptosis, of human HNSCC cell lines derived from the tongue, larynx, oral cavity or pharynx, suggesting that honokiol possesses a potentially broad therapeutic effect in this disease. Moreover, honokiol did not significantly inhibit the proliferation of normal human bronchial epithelial cells (BEAS-2B) [49]. Analysis of Bcl-2 family members [36,37] revealed that the honokiol-induced apoptosis of HNSCC cells (FaDu and SCC-1) was associated with a decrease in the levels of anti-apoptotic proteins and a simultaneous increase in the pro-apoptotic proteins. [35]. Honokiol also affected the cell cycle check points in the FaDu and SCC-1 HNSCC cell lines including G0–G1 phase arrest and a marked decrease in the levels of cyclin D1, D2 and Cdks in. This effects may represent one of the possible mechanisms action of that mediate the anti-carcinogenic effects of honokiol in HNSCC cells (Figure 2). 

Treatment of HNSCC cells with honokiol also decreased the expression of total EGFR as well as p-EGFR and its downstream target, mTOR. As activation of mTOR has been shown to contribute in tumor progression, it can be speculated that the honokiol-induced inhibition of cell proliferation of HNSCC cells is mediated, at least in part, through the downregulation of EGFR/mTOR signaling pathway [51]. These observations are consistent with the evidence that honokiol inhibits the growth of cancer cells by targeting EGFR and its downstream molecular targets and suggest that these mechanism are in play in HNSCC. Notably, molecular docking analyses suggest that honokiol binds to EGFR and that the binding affinity of honokiol for EGFR is even greater than gefitinib, an EGFR inhibitor like erlotinib [51].

The therapeutic effects of honokiol against HNSCC also have been examined using tumor xenograft model. Administration of honokiol by oral gavage inhibited the growth of FaDu and SCC-1 tumor xenografts in athymic nude mice without showing any apparent visible sign of toxicity in the mice. However, in similar studies, other investigators found that honokiol did not show significant inhibition of tumor xenograft growth in athymic nude mice [52]. The discrepancy in the outcomes of these studies may reflect differences in the dose of honokiol or the use of different cell lines (other than FaDu, SCC-1), which may indicate that the effects of honokiol are cell line-specific. Singh et al. [51] found that the inhibitory effects of honokiol on tumor xenograft growth that they observed were associated with the: (i) Inhibition of PCNA, and correction of dysregulation of proteins associated with cell cycle progression; (ii) Induction of apoptotic cell death of tumor cells, as indicated by increased levels of activated caspase-3; and (iii) reduction in the levels of EGFR, pAkt and mTOR ([53], and Figure 2). These studies indicate that honokiol is an attractive bioactive phytochemical for the management of head and neck cancer. They suggest that it could be used either alone or in combination with other available therapeutic drugs to boost their efficacy. Further studies are necessary to identify and verify the molecular targets of honokiol associated with HNSCC specifically and the possibility that its efficacy is associated with subtypes of this malignancy. 

### 2.3. Green Tea Polyphenols and HNSCC

Infusions of the leaves of the tea plant (*Camellia sinensis*) are a popular beverage worldwide because of their characteristic aroma, flavor and health benefits [53,54,55,56]. Of the total commercial tea production worldwide, approximately 80% is in the form of black tea, which is marketed mostly in Western countries, and approximately 20% in the form of green tea. Oolong tea, a partially fermented tea, is favored in some parts of South-eastern China [54,55]. The characteristic aroma and health benefits of green tea are associated with the presence of polyphenols. The major polyphenolic constituents present in green tea are (−)-epicatechin, (−)-epigallocatechin, (−)-epicatechin-3-gallate and (−)-epigallocatechin-3-gallate (EGCG) [54,55,56]. Black tea mainly contains thearubigins and theaflavins, which are polymerized forms of catechin monomers [53,54]. As several in vitro and in vivo studies have indicated that the polyphenols present in green tea are better chemopreventive agents than the polyphenols present in black tea, most investigations have focused on the activity of the green tea polyphenols (GTPs). There is a large body of evidence that green tea polyphenols have antioxidant and anti-inflammatory properties, can repair damaged DNA, and can stimulate the immune system. In addition, the chemopreventive and chemotherapeutic effects of GTPs have been tested in various tumor models [57,58,59,60,61,62,63,64,65,66,67]. EGCG treatment of the HNSCC cell lines, YCU-N861 and YCU-H891, was found to result in induction of apoptosis, which was associated with an increase in arrest of the cell cycle at the G1 phase, a decrease in the levels of cyclin D1 protein, and an increase in the levels of the tumor suppressor proteins Cip1/p21 and Kip1/p27 (Figure 2 and Figure 3) [67]. The treatment with EGCG inhibited phosphorylation of EGFR, signal transducer and activator of transcription 3 (Stat 3), ERK proteins and NF-κB. Additionally, treatment with EGCG reduced the levels of VEGF produced by the HNSCC cells [67,68,69]. Other investigators also have provided evidence supporting the anti-HNSCC effect of EGCG using other biomarkers of interest. These have been reviewed by Masuda et al. [70], who provided evidence that the inhibitory effects of EGCG on HNSCC cells are associated with modulation of signal transduction pathways triggered by signaling through EGFR and other receptor tyrosine kinases. Interestingly, these authors also postulated that the modulation of the signal transduction effects may be attributable to a direct biophysical effect of EGCG on the “lipid rafts” present in cell membranes. Other pathways that have been implicated in the EGCG effects in HNSCC include inhibition of NF-κB signaling [71] and involvement of ATM-dependent p53 expression [72]*.*

Green tea has been used in some clinical trials to test its effects in HNSCC in combination with other natural products, vitamin A and E, and COX-inhibitors. Encouraging outcomes were found with most natural extracts [73]. Other studies have shown that a formulation of EGCG with curcumin and resveratrol inhibited the tumorigenicity of human papillomavirus-positive head and neck SCC [74], that a combination of GTPs and other phytonutrients inhibited human HNSCC associated with Fanconi anemia [75,76]. 

## 3. Bioavailability and Toxicities of GSPs, GTPs and Honokiol

The bioavailability and toxicity of therapeutic phytochemicals is an important issue in all investigations. It has been recognized that polymeric proanthocyanidins are not absorbed as such in the gut [77]. However, proanthocyanidin dimers were detected in human plasma [78,79] but their absorption is significantly lower than that of the monomeric flavanols [78]. These compounds protect against oxidative stress or the actions of carcinogens. It has been found that long-term consumption of GSPs by laboratory animals through supplemented diet did not show apparent toxic effects in animals [50]. In addition, consumption of proanthocyanidin-rich foods, such as red wine and grape seed extracts, have been shown to increase the plasma antioxidant capacity and have positive effects on vascular function in humans [80]. Bioavailability and toxicities of GTPs also have been studied extensively, and they did not show significant toxicities as determined in vitro and in vivo systems [62]. The author’s laboratory has determined whether long-term consumption of GTPs in drinking water of mice has any toxic effects on animal’s health. It was observed that regular consumption of GTPs by mice through drinking water for more than 6 months did not affect the normal growth of their body weight and did not affect the normal consumption of diet and water on per day basis compared to non-GTPs-fed control mice. Further, the long-term effects of GTPs on liver toxicity was also determined. Mice were given either normal drinking water or GTPs (0.2%, *w*/*v*)-containing drinking water for 6 months. At the termination of the experiment, liver specific enzymes/contents, such as aspartate aminotransferase and alkaline phosphatase were determined in the serum samples of mice using standard procedures. It was observed that there was no significant difference in the levels of these enzymes in the serum samples of GTPs-fed and non-GTPs-fed group of mice, suggesting the non-toxic effect of GTPs on liver after its long-term treatment (Katiyar et al., University of Alabama at Birmingham, Birmingham, AL, USA, 2016). In contrast, Church et al. [81] has reported that intake of EGCG (50 mg/kg; daily for three days) exhibited severe hepatotoxicity in Diversity Outbred (a genetically heterogeneous mouse population) mice. The toxicity of EGCG was found in only 16% animals and this toxicity of EGCG was specific to genetic modification of the animals. Attempts have been made to increase the bioavailability of honokiol in in vivo model. Han et al [82] have developed nanosuspensions of honokiol with a mean particle size of 116.2 nm and a high drug payload (50.4% ± 0.6%, *w*/*w*). Nanosuspensions were prepared by using bovine serum albumin and polyvinylpyrrolidone as stabilisers in a solvent precipitation-ultrasonication method. Honokiol nanosuspensions improved the oral bioavailability of honokiol in in vivo studies in rats. Intraperitoneal administration of honokiol nanosuspensions was found better than oral administration in terms of biodistribution of honokiol.

## 4. Conclusions

HNSCC is a challenging clinical problem and it has been recognized that there is a need to develop alternative options for the management of this malignancy and, especially, for prevention of the invasiveness of these tumors. Plant products and phytochemicals, which have been used for thousands of years, may provide novel leads. If, any phytochemical is non-toxic or less toxic than currently available therapeutic strategies and has adequate anti-cancer properties, they should be considered candidates for assessment of their therapeutic efficacy against HNSCC in humans. The current literature analysis summarizes the evidence demonstrating the effects of three promising phytochemicals, GSPs, honokiol and GTPs, in HNSCC. Notably, these phytochemicals, and in particular GSPs and GTPs, are already used routinely as a part of the human diet or as dietary supplements. As described in this communication, GSPs, GTPs/EGCG and honokiol inhibit the growth and cell migration abilities of HNSCC cells. Notably, they all target multiple molecular targets in a manner that is consistent with their anti-carcinogenic effects, as summarized in Figure 2 and Figure 3. Bioavailability and toxicities of GTPs and GSPs have been studied extensively, and they did not show significant toxicities as determined in vitro and in vivo systems [25,62]. Additional studies are required to further define whether these phytochemicals show equivalent effectiveness against HNSCC tumors derived from different subsites. In addition, determination of the efficacy of combinations with other known therapeutic cancer drugs is need to indicate whether they can induce dose-sparing effects and thus minimize toxicities or prevent the development of drug resistance. Currently, the evidence indicates that the use of these phytochemicals, alone or in combination therapies, can be considered as to be a potentially effective strategy for the treatment of head and neck cancers or its prevention in high-risk individuals.
